# Supplementary material for: Maximal response to a mechanical leader at critical group size in ant collectives
Source: Nat Commun. 2025 Jul 1;16:5983. doi: 10.1038/s41467-025-61158-6 (PMC12219395; doi:10.1038/s41467-025-61158-6)
Supplement: Supplementary file 1 — Supplementary information [file 41467_2025_61158_MOESM1_ESM.pdf]

Maximal response to a mechanical leader at critical group size in ant collectives

Atanu Chatterjee* and Tom Tzook

Department of Physics of Complex Systems, Weizmann Institute of Science, Rehovot, Israel, 7610001

Nir Gov

Department of Chemical and BiologicalPhysics, Weizmann Institute of Science, Rehovot, Israel, 7610001

Ofer Feinerman†

Department of Physics of Complex Systems, Weizmann Institute of Science, Rehovot, Israel, 7610001

CANTILEVER DEFLECTION CALIBRATION

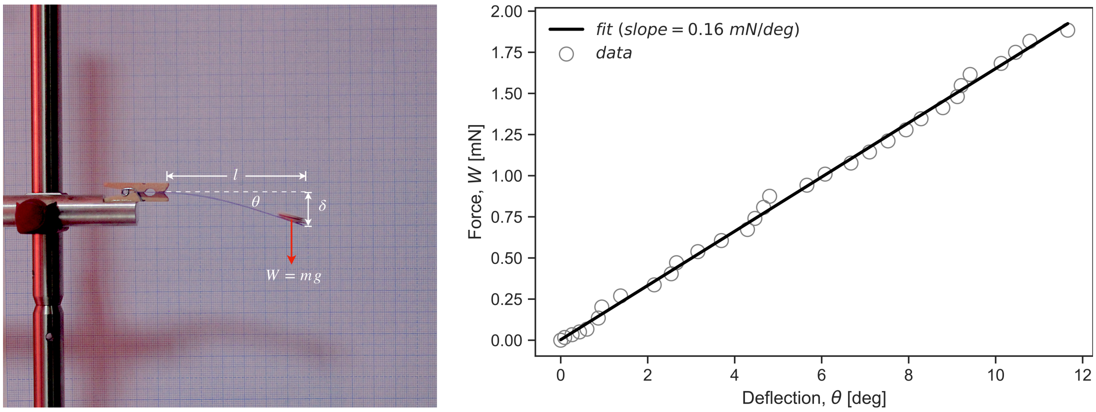

FIG. S1. **Force deflection calibration of the cantilever arm**. Frame recording shows the deflection δ experienced by the cantilever arm under the influence of the point load W (left). The force-deflection curve (W vs. δ) for the cantilever arm is shown on the right.

The cantilever arm is held fixed at one end and loaded with weights (W) at the other, causing it to bend. As additional weights are gradually added, a camera captures the progressive deflection in the arm. Using ImageJ, the deflection (δ) corresponding to each added weight is measured relative to the deflection recorded in the reference image, where $\delta = \delta_0$ represents the initial deflection in the cantilever due to its self-weight, W_0. The angular deflection (θ) is then calculated using the formula $\tan^{-1}(\delta - \delta_0)/l$, where l denotes the length of the cantilever arm from the fixed end to the point of the force application. In Fig. S1, a frame recording of the cantilever calibration experiment is presented, along with the force-deflection curve. In the experiments, the angular deflection in the cantilever is transmitted as a tangential force (F_{ext}) at the cargo end: $F_{\text{ext}} = l\,(k\Delta\theta)\,/l_{\text{rod}}$, where k is the elastic constant of the cantilever. This force counteracts the efforts of the uninformed puller ants, effectively nudging the cargo back toward the nest.

HINGED CARGO DYNAMICS

The hinged cargo is pulled towards the nest at $\theta = 0$ by the collective efforts of the ants. In Fig. S2, we plot the angular velocity as a function of the angular position of the cargo (in the absence of external forces from the robot) for different group sizes. The cargo fluctuates around the nest for the small group size. However, as the group size increases from small to very large, both the amplitude and persistence of these oscillations grow. Thus, the cargo transitions from a disordered state to an ordered state as the group size increases. This transition in the angular velocity distribution is used to quantify the order parameter in the system, as elaborated in the *Main Text*, Fig. 2.

* achatterjee.physics@gmail.com
† ofer.feinerman@weizmann.ac.il

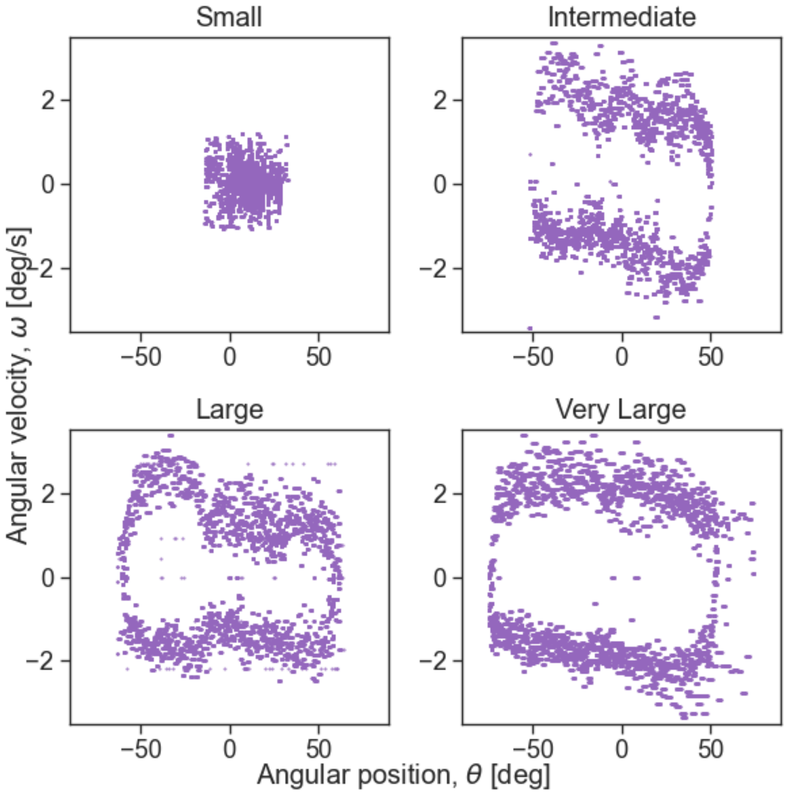

FIG. S2. **Cargo angular position and angular velocity in the absence of any external force.** Figure shows the hinged cargo's angular position and angular velocity as a function of the group size without any external force from the robot. The cargo moves erratically for small group sizes ($N = 2 - 4$ ants) with very low angular velocity and minimal angular change. For the intermediate groups with $N = 15 - 20$ ants, the cargo oscillates with relatively small amplitudes with very low angular velocity. The cargo exhibits persistent oscillations with larger amplitudes in the ordered regime, corresponding to large and very large group sizes ($N = 30 - 40$ and $N = 50 - 60$ ants, respectively).

MICROSCOPIC MODEL

The cargo ring carried by the ants is modeled as a circle with equally spaced sites labeled by an angle $\alpha_i, i \in [1, N_{\max}]$ where $N_{\max}$ is the maximum number of sites on the cargo. A site can be empty or occupied by a puller or a lifter.

- **Initialize the agents**: An ant can be a puller or a lifter. If she is a puller, she can either be an informed or uninformed puller. The arrays for each ant category are of length $N_{\max}$, which is the maximum number of sites on the cargo.
  - **Puller**: array where each element represents whether an ant is a puller (0 for false, 1 for true).
  - **Lifter**: array indicating if an ant is a lifter (0 for false, 1 for true).
  - **Informed**: array denoting if an ant is informed about the direction towards the nest (0 for false, 1 for true).
  - **Phi (ϕ)**: a floating-point array representing the orientation angle of each puller ant. An ant orientation is measured relative to the outward site normal and is limited by $\phi_{\max} = 52°$. As lifters do not contribute to the carrying process, their orientation is not adjusted, and $\phi = 0$.
- **Force calculation**: The force exerted by a puller ant at a cargo site, i is given as, $\mathbf{f}_i = f_0 \hat{p}_i$ where $\hat{p}_i = \cos(\alpha_i + \phi_i) + \sin(\alpha_i + \phi_i)$ is the body axis vector of the ant relative to the outward site normal.
  - the cargo is constrained to move along a circle of fixed radius; therefore, the pulling forces from the puller ants are resolved orthogonal to the cargo center of mass. Therefore, the total force from uninformed pullers, $\mathbf{F}_{\text{puller}} = \left(\left(\sum_i \mathbf{f}_i \right) \cdot \hat{t} \right) \hat{t}$, where $\hat{t}$ is the unit vector orthogonal to the cargo center of mass. At

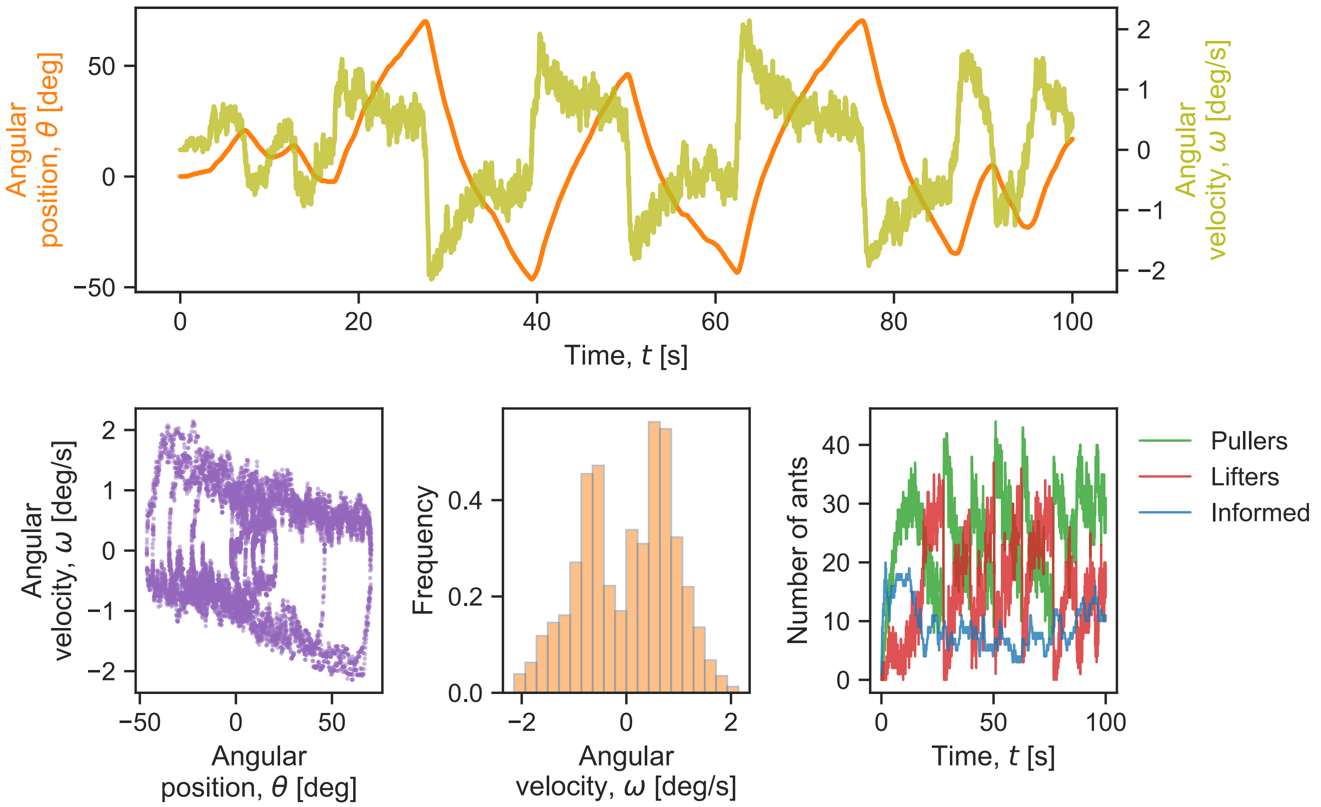

FIG. S3. **A simulation of the ant cargo**. The position velocity plot shows the onset of oscillations in the ant-cargo system in the top panel. The angular position of the cargo is measured relative to the nest at 0°. The $\theta - \omega$ phase portrait, angular velocity distribution, and the number of pullers, lifters, and informed ants in the system are shown. The model parameters used are: $N_{\text{max}} = 60$, $F_{\text{ind}} = 28$, $f_0 = 2.8$, $k_{\text{on}} = 0.021$, $k_{\text{off}} = 0.015$, $k_{\text{forget}} = 0.09$, $k_c = 1$, $k_{\text{ori}} = 0.7$, and $\gamma = 180$. The average number of ants that were attached to the cargo during the entire simulation was $\langle N \rangle = 54$.

any given moment in time, t, the cargo center of mass in polar coordinates is, $(\cos(\theta(t)), \sin(\theta(t)))$; then $\hat{t} = (-\sin(\theta(t)), \cos(\theta(t)))$.

- the pulling forces from the informed ants is resolved along the nest direction at $(1, 0)$, $\mathbf{F}_{\text{informed}} = (\sum_i f_0 \cos(\alpha_i + \phi_i), 0)$.
- The total force is then, $\mathbf{F}_{\text{tot}} = \mathbf{F}_{\text{puller}} + \mathbf{F}_{\text{informed}}$.

• **Initialize the rates**: Initialize the rates of attachment, detachment, reorientation, conversion, and forgetting.

- **Attachment rate (R_{att})**: calculates the rate of ants attaching to the cargo, proportional to the number of empty sites. It is calculated as: $R_{\text{att}} = k_{\text{on}} \times$ Number of empty sites.
- **Detachment rate (R_{det})**: determines the rate of ants detaching from the cargo, proportional to the number of attached ants (both pullers and lifters). It is calculated as: $R_{\text{det}} = k_{\text{off}} \times \sum_i (\text{pullers}_i + \text{lifters}_i)$.
- **Reorientation rate (R_{orient})**: computes the rate at which informed puller ants reorient themselves, proportional to their total number. It is calculated as: $R_{\text{orient}} = k_{\text{ori}} \times \sum_i (\text{pullers}_i + \text{informed}_i)$.
- **Conversion rate (R_{con})**: computes the conversion rate between puller and lifter ants. It is given by, $R_{\text{con}} = k_c \times \sum_i (\text{pullers}_i \times \exp(-\mathbf{f}_{\text{loc}} \cdot \hat{p}_i / F_{\text{ind}}) + \text{lifters}_i \times \exp(\mathbf{f}_{\text{loc}} \cdot \hat{p}_i / F_{\text{ind}}))$, where the orientation of puller/lifter is $\hat{p}_i$ and the local force $\mathbf{f}_{\text{loc}}$. In a solid cargo with strongly coupled ants, $\mathbf{f}_{\text{loc}} = \mathbf{F}_{\text{tot}}$.
- **Forgetting rate (R_{forget})**: calculates the forgetting rate proportional to the number of informed ants. It is calculated as: $R_{\text{forget}} = k_{\text{for}} \times \sum_i \text{informed}_i$.

• **Agent interaction rules**: The following rules implement the interaction between the ant and the cargo and also determine the ant behavior throughout the simulation.

- **Attach**: attaches an informed ant to the cargo, making it an informed puller and reorienting it according to the total force towards the nest.
- **Detach**: detaches an ant from the cargo, removing its status as a puller or a lifter, and sets its orientation to NaN (not a number).
- **Reorient**: adjusts the orientation (ϕ_i) of an ant based on its role. For a puller ant, the function calculates the direction of the total force acting on the cargo and aligns the ant orientation to this force direction. For an informed ant, the orientation is adjusted towards the nest.
- **Exponential Rate**: calculates an exponential rate for an ant at a site based on the dot product of the total force vector and the ant orientation vector at a site. This rate influences the likelihood of the ant choosing to pull or lift. It is given as: $\exp\left(-\mathbf{F}_{\text{tot}} \cdot \hat{p}_i / F_{\text{ind}}\right)$, where the orientation of puller/lifter is $\hat{p}_i$ and the total force on the cargo, $\mathbf{F}_{\text{tot}}$.
- **Pull or Lift**: decides whether an ant, after forgetting becomes a puller or a lifter based on a probability influenced by force at that site. This decision is made through a random process by drawing a random number, r_3 from a uniform distribution, $\mathcal{U}(0,1)$. If $r_3 < 1/\left(1 + \exp\left(-\mathbf{F}_{\text{tot}} \cdot \hat{p}_i / F_{\text{ind}}\right)\right)$ then an ant becomes an uninformed puller, else she is a lifter.
- **Convert**: changes the role of an ant from Puller to Lifter or vice versa based on the **Exponential Rate** at a given site. If the ant becomes a puller due to conversion, it reorients to align with the cargo force direction or towards the nest.
- **Forget**: an informed ant forgets, and based on the **Pull or Lift** function, she decides to either pull or lift.

• **Gillespie algorithm and state update**: The time step dt of the next event is calculated by drawing a random number r_1 from a uniform distribution, $\mathcal{U}(0,1)$ such that, $dt = (-1/R_{\text{total}}) \times \log(r_1)$, where R_{total} is the sum of all possible rates given by, $R_{\text{total}} = (R_{\text{att}} + R_{\text{det}} + R_{\text{con}} + R_{\text{orient}} + R_{\text{forget}})$. A random number r_2 is drawn from a uniform distribution, $\mathcal{U}(0,1)$, and the Gillespie steps at time $t + dt$ are determined based on the following conditions.

- if $r_2 < R_{\text{att}}/R_{\text{total}}$ an informed ant attaches to the cargo
- else if $R_{\text{att}}/R_{\text{total}} \leq r_2 < \left(R_{\text{att}} + R_{\text{det}}\right)/R_{\text{total}}$ an uninformed ant detaches from the cargo
- else if $\left(R_{\text{att}} + R_{\text{det}}\right)/R_{\text{total}} \leq r_2 < \left(R_{\text{att}} + R_{\text{det}} + R_{\text{con}}\right)/R_{\text{total}}$ an uninformed ant switches from puller to lifter and vice versa
- else if $\left(R_{\text{att}} + R_{\text{det}} + R_{\text{con}}\right)/R_{\text{total}} \leq r_2 < \left(R_{\text{att}} + R_{\text{det}} + R_{\text{con}} + R_{\text{orient}}\right)/R_{\text{total}}$ an ant orients its position with respect to the total force vector
- else $R_{\text{forget}} \neq 0$ an informed ant turns into an uninformed ant

• **State update**: The ant-cargo system is over-damped. Therefore the force is proportional to velocity, $\mathbf{F}_{\text{tot}} = \gamma \mathbf{v}$, where γ is the damping coefficient. The cargo position is obtained by numerically integrating the cargo velocity over the step size. The stochastic update (dt) determines when the next event occurs based on the current state and rates of possible events. The method of dividing dt into segments of a fixed step size (0.01 s) to update the system, especially the cargo position, integrates the effects of continuous forces and movements within the intervals defined by stochastic events. This integration respects the continuous nature of cargo mechanics within the constraints imposed by discrete, stochastic events. In Fig. S3, cargo oscillations are shown at the onset of the simulation. Also shown in the bottom panel are the phase portrait, the number of pullers, lifters, and informed ants, and the angular velocity distribution.

• **Boundary condition**: The boundary conditions ensure that the origin of the rod is fixed at (0,0) and the cargo is constrained to rotate along a circle of fixed radius (l_{rod}).

• **External force application**: After 10000 simulation time steps (100 s) have elapsed, an external force, $\mathbf{F}_{\text{ext}}$ over Δt duration is applied to the cargo to simulate the influence of the robot as a transient leader. Its direction is based on the current position of the cargo: first, it is ensured if the cargo has crossed the nest, and second, if the cargo moves clockwise, then the force is applied counter-clockwise and vice versa. The implementation of the forcing function is via a hyperbolic tangent function $F_{\text{ext}}\left(\tanh(t - t_{\text{start}}) - \tanh(t - t_{\text{end}})\right)$ where $t_{\text{end}} = t_{\text{start}} + \Delta t$.

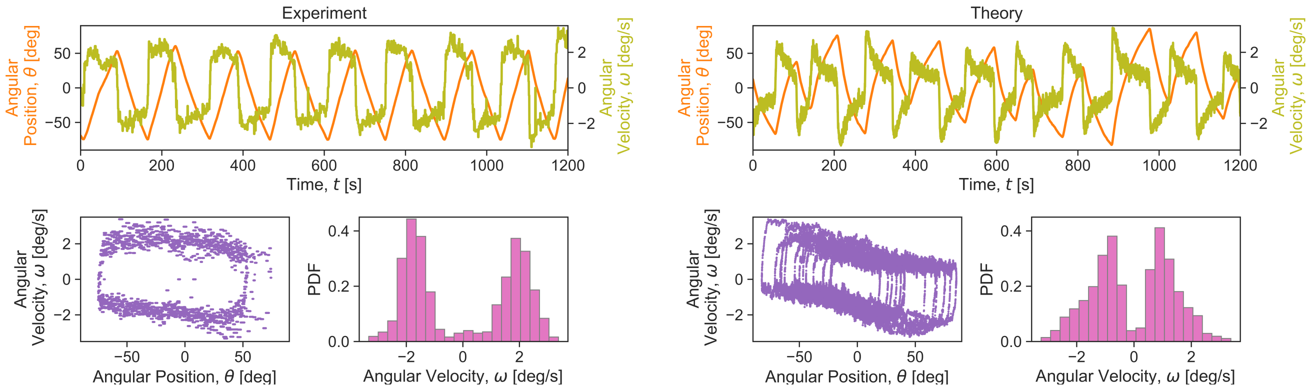

FIG. S4. **Experiment and theory for the largest group size**. (left) Empirical data shows periodic oscillations in the largest group size ($N = 50 - 60$ ants) in the absence of any external forcing. (right) Numerical simulation from the theoretical model replicates the behavior and is used to calibrate the model parameters. For the simulation the following parameters were used: $F_{\text{ind}} = 28$, $f_0 = 2.8$, $k_{\text{on}} = 0.021$, $k_{\text{off}} = 0.015$, $k_{\text{forget}} = 0.09$, $k_c = 1$, $k_{\text{ori}} = 0.7$. The maximum number of attachment sites was also set to $N_{\text{max}} = 60$, with a damping coefficient of $\gamma = 180$.

PARAMETER ESTIMATION

There are numerous parameters that the microscopic model utilizes, such as the number of ants (N_{max}), scale ant force (f_0), individuality parameter (F_{ind}), damping coefficient (γ), and several variables that capture basal rates of attachments (k_{on}), detachments (k_{off}), forgetting (k_{forget}), and decision-making (k_c), the only parameter that is changed group to group is the number of ants. The damping coefficient scales with group size, i.e., cargo diameter and the basal rates, are kept unchanged for all the simulations. The rates k_{off}, k_{for}, k_c, and k_{ori} remain unchanged from previous studies [1]. However, we increased the value of k_{on} to ensure that the cargo is always fully saturated with ants, mirroring our experimental observations. To account for the intermittent “jerks” that the agents (ants) experience when the robot nudges the cargo in experiments, we also increased the individuality parameter F_{ind} to 28, compared to the value of 10 used in earlier works [1], because larger F_{ind} corresponds to more noisy ant behavior. The maximum number of attachment sites N_{max} varies with each group, and the damping coefficient γ scales linearly with N_{max}. Finally, we calibrated the model using the angular velocity and oscillation amplitude measured from the experiments for each cargo. In Fig. S4, we present the empirical data on the left panel and the theory data on the right. The model parameters used in this case are: $F_{\text{ind}} = 28$, $f_0 = 2.8$, $k_{\text{on}} = 0.021$, $k_{\text{off}} = 0.015$, $k_{\text{forget}} = 0.09$, $k_c = 1$, $k_{\text{ori}} = 0.7$. For the largest group size, $N_{\text{max}} = 60$ and damping coefficient $\gamma = 180$.

MEAN-FIELD APPROXIMATION

This section discusses the mean-field model when subjected to the external force, $F(t)$. In Fig. S5, a schematic of the ant-cargo pendulum is shown. The cargo is held by a stiff rod that is free to rotate about a pivot. The pullers (green ants) apply a tangential force, while the lifters (red ants) minimize friction. The influence of the nest is due to the informed ants that carry directional information of the nest and are represented by G. The total force experienced by the cargo is given by,

$$F_{\text{tot}} = f_0 n_p^{\text{front}} - f_0 n_p^{\text{rear}} - f_0 G \sin\theta - F(t) \tag{1}$$

In the given equation, f_0 represents the force exerted by a single puller ant, G stands for the number of informed ants, and n_p denotes the number of puller ants. The rates of transition between pulling and lifting are determined by $r_{p\to l}^{\text{front/rear}} = k_c \exp\left(\mp F_{\text{tot}}/F_{\text{ind}}\right)$ and $r_{l\to p}^{\text{front/rear}} = k_c \exp\left(\pm F_{\text{tot}}/F_{\text{ind}}\right)$. The orientation of the puller ants at the front and rear, in relation to the mirror symmetry of the circular cargo, is based on the direction of motion of the cargo. Upon taking the time derivative of the equation above,

$$\frac{dF_{\text{tot}}}{dt} = f_0\left(\frac{dn_p^{\text{front}}}{dt} - \frac{dn_p^{\text{rear}}}{dt}\right) - f_0 G(\cos\theta)\frac{d\theta}{dt} - \frac{dF(t)}{dt} \tag{2}$$

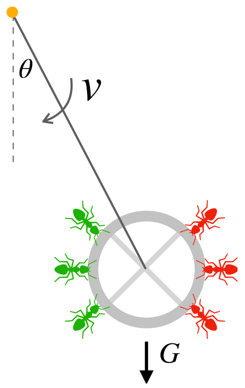

FIG. S5. **A mean-field ant-cargo pendulum**. A schematic representation of the ant-cargo pendulum system undergoing oscillation is shown. The green ants are the pullers, and the red ants are the lifters. The influence of the informed ants is represented by G.

The pulling effort depends on the number of pullers on either side of the cargo. The rate of change in the number of pullers on each side can be described by the following equations:

$$\left(\frac{dn_p}{dt}\right)^{\text{front}} = \left(r_{l\to p}n_l - r_{p\to l}n_p\right)^{\text{front}} \quad \text{and} \quad \left(\frac{dn_p}{dt}\right)^{\text{rear}} = \left(r_{l\to p}n_l - r_{p\to l}n_p\right)^{\text{rear}} \tag{3}$$

The total number of uninformed ants connected to the cargo is denoted as N. Among the $N/2$ ants on each side, which represent the uninformed population, the pullers and lifters satisfy the relationships $n_p^{\text{front}} + n_l^{\text{front}} = N/2$ and $n_p^{\text{rear}} + n_l^{\text{rear}} = N/2$. By substituting the rate terms $r_{p\leftrightarrow l}$ into Eqn. 3, we can further analyze the system [2],

$$\left(\frac{dn_p}{dt}\right)^{\text{front}} = k_c\frac{N}{2}\exp\left(\frac{F_{\text{tot}}}{F_{\text{ind}}}\right) - 2k_c n_p^{\text{front}}\cosh\left(\frac{F_{\text{tot}}}{F_{\text{ind}}}\right) \tag{4}$$

$$\left(\frac{dn_p}{dt}\right)^{\text{rear}} = k_c\frac{N}{2}\exp\left(-\frac{F_{\text{tot}}}{F_{\text{ind}}}\right) - 2k_c n_p^{\text{rear}}\cosh\left(\frac{F_{\text{tot}}}{F_{\text{ind}}}\right) \tag{5}$$

Subtracting the rate of change of pullers in the front and rear from the above equation and substituting the difference in Eqn. 2 we have,

$$\frac{dF_{\text{tot}}}{dt} = k_c N f_0 \sinh\left(\frac{F_{\text{tot}}}{F_{\text{ind}}}\right) - 2k_c(n_p^{\text{front}} - n_p^{\text{rear}}) f_0 \cosh\left(\frac{F_{\text{tot}}}{F_{\text{ind}}}\right) - f_0 G\cos\theta\frac{d\theta}{dt} \tag{6}$$

We know from Eqn. 1, $n_p^{\text{front}} - n_p^{\text{rear}} = \left(F_{tot} + f_0 G\sin\theta + F(t)\right)/f_0$. We substitute this back into the above expression and rewrite it,

$$\frac{dF_{\text{tot}}}{dt} = k_c N f_0 \sinh\left(\frac{F_{\text{tot}}}{F_{\text{ind}}}\right) - 2k_c(F_{\text{tot}} + f_0 G\sin\theta + F(t))\cosh\left(\frac{F_{\text{tot}}}{F_{\text{ind}}}\right) - f_0 G\cos\theta\frac{d\theta}{dt} - \frac{dF(t)}{dt} \tag{7}$$

As the system is over-damped, $F_{\text{tot}} = \gamma v$, where γ is the damping coefficient. Thus, dividing the above equation by γ throughout and rearranging the terms we are left with,

$$\frac{1}{\gamma}\frac{dF_{\text{tot}}}{dt} = k_c\frac{Nf_0}{\gamma}\sinh\left(\frac{F_{\text{tot}}/\gamma}{F_{\text{ind}}/\gamma}\right) - 2k_c\left(\frac{F_{\text{tot}} + f_0 G\sin\theta + F(t)}{\gamma}\right)\cosh\left(\frac{F_{\text{tot}}/\gamma}{F_{\text{ind}}/\gamma}\right) - \frac{f_0 G}{\gamma}\cos\theta\frac{d\theta}{dt} - \frac{1}{\gamma}\frac{dF(t)}{dt} \tag{8}$$

The terms, $(f_0 G/\gamma, f_0 N/\gamma, F_{\text{ind}}/\gamma)$ are relabelled as, $\left(\tilde{G}, \tilde{N}, \tilde{F}_{\text{ind}}\right)$, and substituted back,

$$\frac{dv}{dt} = k_c\tilde{N}\sinh\left(\frac{v}{\tilde{F}_{\text{ind}}}\right) - 2k_c\left(v + \tilde{G}\sin\theta + \frac{F(t)}{\gamma}\right)\cosh\left(\frac{v}{\tilde{F}_{\text{ind}}}\right) - \tilde{G}\cos\theta\frac{d\theta}{dt} - \frac{1}{\gamma}\frac{dF(t)}{dt} \tag{9}$$

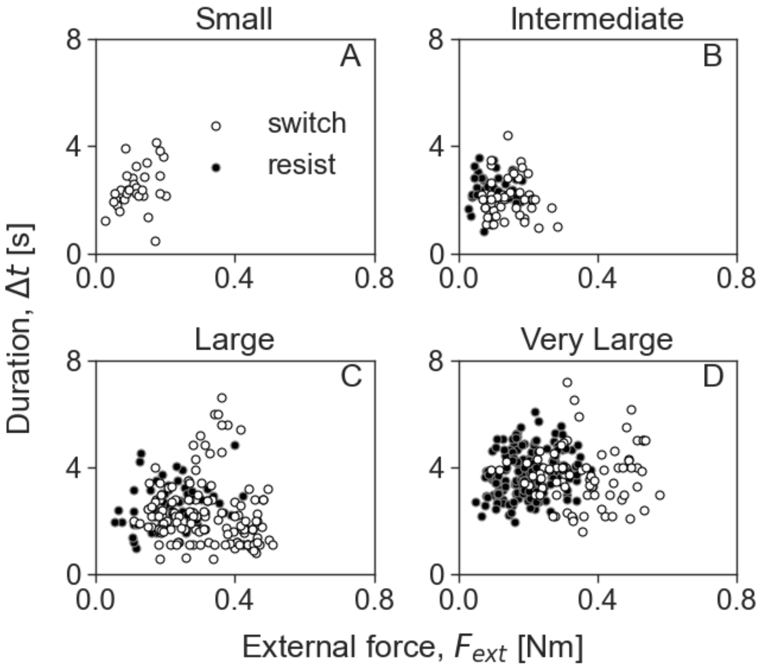

FIG. S6. **Collective response to external forces:** White scatter points represent the instances in experiments when the group switched. In contrast, black scatter points denote the cases where the group resisted a combination of $(\Delta t, F_{\text{ext}})$. The empirical data consists of multiple trials for each group size: (**A**) $n = 31$ for small, (**B**) $n = 85$ for intermediate, (**C**) $n = 201$ for large, and (**D**) $n = 260$ for very large groups.

As the cargo is constrained to move along a circular path of radius, l_{rod} the angular velocity, $d\theta/dt = v/l_{\text{rod}}$. Thus the set of differential equations that describe the mean-field ant-cargo system is given by,

$$\frac{d\theta}{dt} = \frac{v}{l_{\text{rod}}} \tag{10}$$

$$\frac{1}{k_c}\frac{dv}{dt} = \tilde{N}\sinh\left(\frac{v}{\tilde{F}_{\text{ind}}}\right) - 2\left(v + \tilde{G}\sin\theta + \frac{F(t)}{\gamma}\right)\cosh\left(\frac{v}{\tilde{F}_{\text{ind}}}\right) - \frac{\tilde{G}}{k_c l_{\text{rod}}} v\cos\theta - \frac{1}{\gamma}\frac{dF(t)}{dt} \tag{11}$$

The above equation is numerically solved by introducing an external force in the form of a short rectangular pulse. The pulse is introduced when the cargo points toward the nest, located at $\theta = 0°$, at $t = t'$ over a duration Δt. Thus, $F(t) = F_{\text{ext}}$ for $t' \le t \le t' + \Delta t$, and 0 otherwise. As a result, the velocity equation above can be expressed using Heaviside and Delta function notation as shown below,

$$\begin{aligned} \frac{1}{k_c}\frac{dv}{dt} = &\frac{f_0 N}{\gamma}\sinh\left(\frac{v}{F_{\text{ind}}/\gamma}\right) - 2\left(v + \frac{f_0 G}{\gamma}\sin\theta\right)\cosh\left(\frac{v}{F_{\text{ind}}/\gamma}\right) - \frac{f_0 G}{\gamma}\frac{1}{k_c}\frac{v\cos\theta}{l_{\text{rod}}} \\ &- \frac{2F_{\text{ext}}}{\gamma}\cosh\left(\frac{v}{F_{\text{ind}}}\right)\left(H(t-t') - H(t-(t'+\Delta t))\right) \\ &- \frac{F_{\text{ext}}}{\gamma}\left(\delta(t-t') - \delta(t-(t'+\Delta t))\right) \end{aligned} \tag{12}$$

EXPERIMENTS WITH THE ROBOT

We apply ant-scale forces to various cargo sizes and record the collective response. In Fig. S6, the experimental data is represented as scatter points, indicating whether the cargo switches direction or resists the external forces

applied to it. The total duration of the force application is defined as $\Delta t = (t_{\text{end}} - t_{\text{begin}})$, where t_{begin} marks the time when the force application begins and t_{end} marks when it concludes. However, if the group aligns its direction of motion with the applied force, the switching time (t_{switch}) is determined from the cargo's angular velocity time series as the moment when its angular velocity changes direction. In this case, the duration is defined as the time required for the group to switch and align with the force, calculated as $\Delta t = (t_{\text{switch}} - t_{\text{begin}})$.

ROBOT PSEUDO CODE

Algorithm 1 Cargo tracking and force application by the robot. The algorithm presents a schematic of the algorithm that controls the robot to constantly track the cargo and apply a small force through angular deformation, θ of the cantilever arm over a short duration, Δ by nudging the cargo towards the nest.

```
function MotorToAnt(offset)
    Move motor to new position by adding offset
end function
function Force(θ, Δ)
    Move motor by angle θ
    Wait for Δ seconds
end function
function PushTime(Δ)
    Move motor at a constant speed
    Wait for Δ seconds
    Cancel the movement
end function
function PushDegrees(θ)
    Move motor to a new position by angle θ
    Wait until movement completes
end function
initialOffset ← some value
MotorToAnt(initialOffset)
Print "reached flag"
while true do                                          ▷ Continuous tracking and adjusting
    if condition to follow cargo is met then
        Force(θ, Δ)                                    ▷ Apply force as needed
    else if specific condition for push time is met then
        PushTime(Δ)                                    ▷ Push for a specified time
    end if
    Update conditions based on cargo's proximity to nest and other factors
    Sleep for a short duration to simulate time passing
end while
Print "script end"
```

An algorithm is designed to enable a robot to track the cargo in a closed loop consistently. This algorithm dynamically adjusts the position of the blade using a set of defined functions, each customized for specific movement and control tasks. Subsequently, the stepper motor is fine-tuned based on a predefined offset, which indicates the location of the cantilever arm in relation to the blade position and the two vertical screws. The robot applies force to the cantilever arm by rotating the motor to a specified angle (θ) for a specific duration (Δ), initiating the cantilever engagement and causing the screw to exert force, resulting in the bending of the cantilever. Once the predetermined force has been applied for the specified duration, the motor retraces its path to ensure precise positioning.

[1] A. Gelblum, I. Pinkoviezky, E. Fonio, A. Ghosh, N. Gov, and O. Feinerman, Ant groups optimally amplify the effect of transiently informed individuals, Nature communications **6**, 7729 (2015).

[2] A. Gelblum, I. Pinkoviezky, E. Fonio, N. S. Gov, and O. Feinerman, Emergent oscillations assist obstacle negotiation during ant cooperative transport, Proceedings of the National Academy of Sciences **113**, 14615 (2016).
