# Supplementary material for: Maximal response to a mechanical leader at critical group size in ant collectives
Source: Nat Commun. 2025 Jul 1;16:5983. doi: 10.1038/s41467-025-61158-6 (PMC12219395; doi:10.1038/s41467-025-61158-6)
Supplement: Supplementary file 3 — Description of Additional Supplementary Files [file 41467_2025_61158_MOESM3_ESM.pdf]

Description of Additional Supplementary Files

File Name: Supplementary Movie 1

Description: Robot perturbing cooperative cargo transport while the cargo resists.

File Name: Supplementary Movie 2

Description: Robot perturbing cooperative cargo transport and the cargo switches.
